# Exercise adherence and suicidal ideation of Chinese college students: a chain mediation model test

**DOI:** 10.3389/fpsyg.2023.1138469

**Published:** 2023-05-09

**Authors:** Zhi Xing, Kelei Guo, Zhen Hui, Qishuai Ma

**Affiliations:** ^1^School of Physical Education and Health, Zhaoqing University, Zhaoqing, China; ^2^School of Marxism, Zhaoqing University, Zhaoqing, China

**Keywords:** exercise persistence, meaning in life, internet addiction, suicidal ideation, college student

## Abstract

**Objective:**

The purpose of this study was to explore the relationship between exercise adherence and suicidal ideation in college students, as well as the mediating role of meaning in life and internet addiction.

**Methods:**

A total of 1925 college students (*M*_age_ = 19.51 years, *SD*_age_ = 2.393 years) were recruited by stratified cluster sampling method in Zhaoqing University, among which 890 were males and 1,035 were females. Exercise adherence, meaning in life, internet addiction and suicidal ideation were assessed by using standard scales. Data were analyzed by Pearson Correlation Analysis, and bias-correction percentile Bootstrap method.

**Results:**

(1) There is a significant correlation between exercise adherence, meaning in life, internet addiction and suicide ideation; (2) meaning in life plays a significant mediating role between exercise adherence and suicidal ideation; internet addiction plays a significant mediating role between exercise adherence and suicidal ideation; meaning in life and internet addiction play a chain mediating role between exercise adherence and suicide ideation.

**Conclusion:**

Exercise adherence can not only directly predict college students’ suicidal ideation, but also indirectly predict college students’ suicidal ideation through the independent mediation and chain mediation of meaning in life and internet addiction.

## Introduction

Suicide has become an important public health problem around the world (Wang et al., 2019), it is the primary cause of abnormal death among Chinese college students, and their suicide rate is 2–4 times that of the normal population (Liu et al., 2014). An American mental health institute divides suicidal behavior into three steps, namely, suicidal ideation, suicide attempt and suicide death (Shang et al., 2008), among them, suicidal ideation is the first step to commit suicidal behavior, which can accurately predict suicidal behavior (Chao, 2018). Suicide ideation refers to the idea or motive of ending one’s own life that an individual experiences accidentally. It plays an important role in predicting suicide plan and suicide attempt, and it is also the inevitable psychological activity of suicide death in the early stage (Liu and Wang, 2019). Studies have shown that exercise adherence can negatively predict suicidal ideation in college students (Gao and Ren, 2019). Therefore, this study explores the relationship between exercise adherence and suicidal ideation in college students, and also explores whether meaning in life and internet addiction have mediating effects between them, so as to provide theoretical guidance for the prevention of suicide in college students by improving exercise adherence.

### Exercise adherence and suicidal ideation

Exercise adherence refers to the extent to which an individual can effectively achieve a predetermined exercise plan during physical exercise (Wang et al., 2018). Studies have shown that exercise adherence is negatively correlated with suicidal ideation in college students (Brown, 2005; Lei et al., 2021), and exercise adherence can negatively predict suicidal ideation (Wei, 2018). College students with high exercise adherence have lower probability of suicidal ideation (Wang et al., 2022), on the contrary, college students with low exercise adherence had a higher probability of suicidal ideation (Mirim et al., 2008). A longitudinal study has shown that exercise adherence is an important factor in reducing suicidal ideation in college students, and improving exercise adherence can reduce suicidal ideation (Wei and Yang, 2020). Therefore, we propose the following hypothesis:

*Hypothesis 1*: exercise adherence can significantly negatively predict college students’ suicidal ideation.

### The mediating role of meaning in life

Meaning in life refers to the individual’s feeling of life and the individual’s view on the purpose, direction and attitude of life. It is a kind of high-level psychological feeling, including two independent and complementary dimensions: the sense of having meaning and the sense of seeking meaning (Tang, 2008). It is found that the meaning in life may be an important factor in preventing suicidal ideation, and there is a significant negative correlation between the meaning in life and suicidal ideation in college students (Heisel and Flett, 2016; Zhang et al., 2022). The meaning in life can negatively predict suicidal ideation, and improving the meaning in life may help alleviate suicidal ideation (Jose and Angelina, 2019). According to the Stress-susceptibility Model, factors affecting suicidal ideation are mainly divided into protective factors and risk factors (Su et al., 2015). Protective factors such as meaning in life and positive coping style can reduce suicidal ideation (Chao et al., 2007). When people have a stronger meaning in life, they can experience more happiness and happiness, be more optimistic about the future, and have better psychological and social adjustment, which can effectively reduce the risk of suicidal ideation (Zhang and Li, 2018). According to the theory of meaning in lifey, self-awareness is an important cognitive basis for experiencing the meaning of life. When people understand themselves, the world and the relationship between themselves and the world, they can confirm the value of self-existence and thus experience the meaning in life. On the contrary, a sense of emptiness may arise, and even lead to suicidal ideation and behavior (Liu et al., 2013). Therefore, meaning in life may predict suicidal ideation in college students.

In addition, some studies have shown that college students’ exercise adherence is significantly positively correlated with their meaning in life (Liu, 2012), and exercise adherence can positively predict their meaning in life (Yu, 2018). The higher the level of college students’ exercise adherence, the higher the level of their meaning in life (Ding et al., 2016). The longitudinal research have shown that increasing exercise adherence improves adolescents’ meaning in life (Wang et al., 2020). From the perspective of biological mechanism, maintaining physical exercise can affect cognitive aging by reshaping certain brain structures, promoting activation of brain regions related to cognitive function and the connection of functional networks, and can also affect brain function by improving the efficiency of neural processing, thus indirectly affecting psychological experience. Improve individual’s understanding and grasp of life (Zhang and Gao, 2014). In addition, positive psychology believes that a better emotional state generated during physical exercise has an obvious effect on maintaining physical and mental health, and can stimulate and improve self-efficacy, which can affect the choice, duration, effort level and emotional state of an individual’s behavior, to a certain extent, maintain the mood of an individual and stimulate students’ positive and enterprising attitude. Finally, it can improve the sense of meaning of life (Zeng and Zhu, 2021). Therefore, we infer that exercise adherence may predict college students’ meaning in life. To sum up, we propose the following hypothesis:

*Hypothesis 2*: meaning in life plays an mediating role between exercise adherence and suicide ideation.

### The mediating role of internet addiction

Internet addiction is a kind of pathological internet use behavior, which refers to a chronic state of addiction caused by repeated use of the internet, accompanied by increased tolerance and other psychological symptoms of addiction (Cao et al., 2022). Studies have shown that internet addicts have psychological disorders such as depression, loneliness and anxiety, and people with moderate depression are more likely to form internet addiction (Young, 2009). Research has confirmed that exercise adherence is an important factor in predicting college students’ internet addiction, and improving college students’ exercise adherence can effectively reduce internet addiction (Liu, 2018). The study found that most internet addicts lack physical exercise (Cheng, 2009). A meta-analysis shows that exercise adherence can play an intervention role in college students’ internet addiction (Wu et al., 2018). An intervention experimental study showed that long-term physical exercise can significantly reduce the degree of college students’ internet addiction (Zhang and Xu, 2022). From the perspective of biological mechanism, physical exercise can change the content of some neurotransmitters in the body, such as dopamine, 5-hydroxytryptamine and other monoamines as well as endorphins. At the same time, it can stimulate brain function at a wider level, so that individuals can better regulate negative emotions and generate positive emotions, improve loneliness, anxiety and other psychological conditions, promote mental health, and reduce internet addiction (Archer et al., 2015). Sports psychology shows that physical exercise has psychological benefits such as enhancing self-efficacy, reducing stress response and improving individual emotional state, and these positive psychological benefits may have an inhibitory effect on suicidal ideation (Gao and Ren, 2019). Therefore, we infer that exercise adherence can negatively predict college students’ internet addiction.

A large number of studies have proved that there is a certain correlation between internet addiction and suicidal ideation in college students (Wang and Meng, 2014), internet addiction and suicidal ideation in college students are significantly positively correlated (Wen et al., 2021), and internet addiction can positively predict suicidal ideation (Shi and Xie, 2019). The more serious the internet addiction is, the higher the suicidal ideation is, especially the female college students with internet addiction have a higher rate of suicidal ideation (Huang, 2018). An experimental study showed that college students with a high degree of internet addiction had a higher frequency of suicidal ideation than the control group (Fu et al., 2010). According to the Interpersonal Theory of Suicide proposed by Van et al. (2010), interpersonal psychology can predict and reveal suicidal behavior. But under the influence of internet addiction, college students will have bad social relations, mental health and other problems (Odacı and Çelik, 2013). According to the Stress-susceptibility Model of Suicide (Morgan, 2002), suicide is the result of the combination of individual diathesis and external stimuli. In the period of drastic changes in body and mind, college students with prominent rebellious psychology are prone to have impulsive thoughts including suicidal ideation under the stimulation of internet addiction. Therefore, we conclude that internet addiction can positively predict suicidal ideation. Finally, we propose the following hypothesis:

*Hypothesis 3*: internet addiction plays an mediating role between exercise adherence and suicide ideation.

### The chain mediating effect of meaning in life and internet addiction

Studies have found that meaning in life is closely related to internet addiction (Ge et al., 2018), meaning in life can negatively predict internet addiction in college students (Chen et al., 2019a; Cao et al., 2022), loss of meaning in life is an important cause of internet addiction in college students (Zhao et al., 2020). According to the Psychological Needs Theory of Internet Addiction, internet addiction is the result of individual psychological needs not being met in real life and seeking individual psychological needs through the internet (Liu et al., 2016). Individuals with long-term loss of meaning in life cannot meet their psychological needs in reality, and they are more likely to get “pathological compensation” through excessive use of the internet, which leads to internet addiction. At the same time, studies have found that individuals who use the internet excessively will also cause loss of meaning in life (Ge et al., 2018). There may be a two-way correlation between meaning in life and internet addiction. Once out of the internet world, it is easy to cause the loss of meaning in life, over and over again, the individual will aggravate the internet addiction, and then appear more serious psychological problems. Therefore, we propose the following hypothesis:

*Hypothesis 4*: meaning in life and internet addiction play a chain mediating role between exercise adherence and suicidal ideation.

**Figure 1:**
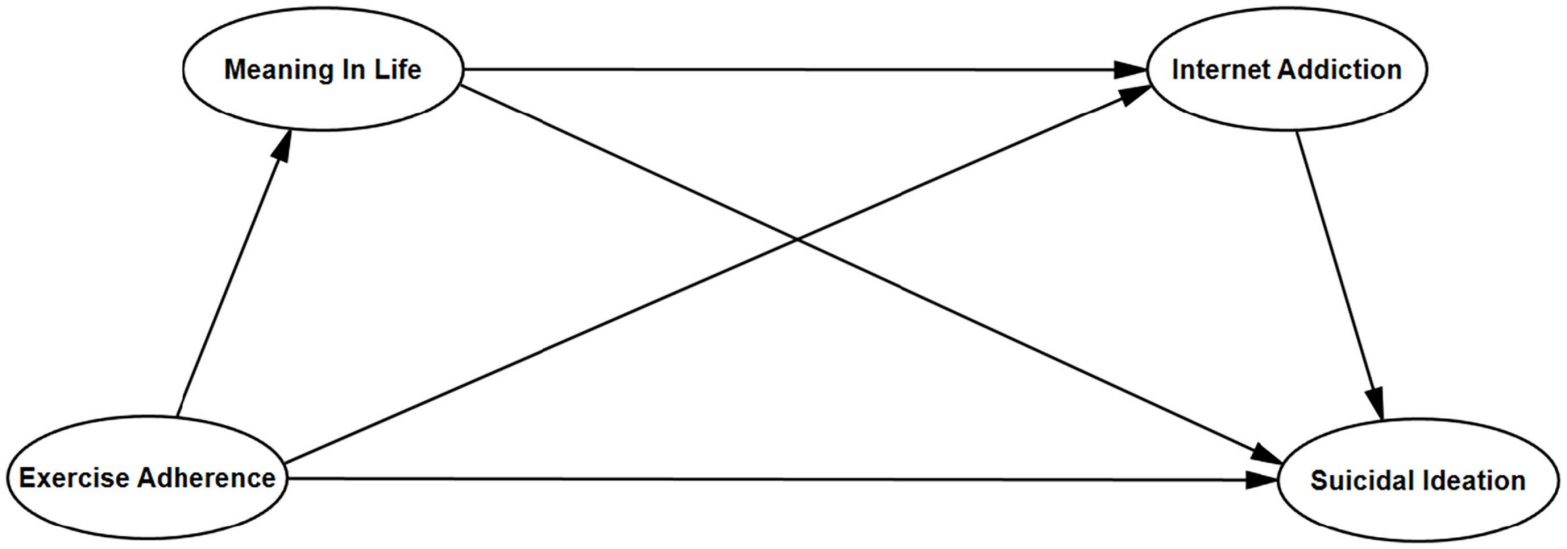
Concept framework.

In summary, this study has four main purposes: (1) To test the negative predictive effect of exercise adherence on college students ‘suicidal ideation; (2) To test the mediating role of meaning in life between exercise adherence and suicidal ideation; (3) To test the mediating effect of internet addiction between exercise adherence and suicidal ideation; (4) To test the chain mediating effect of meaning in life and internet addiction between exercise adherence and suicidal ideation (Figure 1).

## Materials and methods

### Procedure and participants

The stratified cluster sampling was used to select 2,102 undergraduate students from a university in Guangdong Province to complete the questionnaire survey. The invalid questionnaires with regular answers were deleted, and 1,925 valid questionnaires were obtained, with an effective recovery rate of 91.6%. The age range of the subjects was 18–21 years old, with an average age of 19.51 ± 2.393, including 890 boys (46.23%) and 1,035 girls (53.77%). There were 503 freshmen, 406 sophomores, 512 juniors and 504 seniors.

This study was approved by the Research Ethics Committee of Zhaoqing University (No. 2022-1207-01). The collective test was adopted. Before the survey, the students were explained that the questionnaire was anonymously answered, emphasizing voluntary filling. The content is strictly confidential, and the results are only for scientific research. All questionnaires were collected on the spot. All subjects were informed of the purpose and characteristics of the study and signed informed consent. The experimenters were professionally trained college students.

### Measures and instruments

#### Exercise adherence

The Exercise Adherence Scale revised by Liu et al. (2011) was used to evaluate exercise adherence. The scale is based on the theory of sports commitment, a total of 6 items (e.g., participating in physical exercise has become a habit for me), using Likert 5-point evaluation, 1 represents completely disagree, 5 represents completely agree. The total score represents the level of exercise adherence. The higher the score, the higher the level of sports adherence. The research proves that the questionnaire has good applicability in college students (Dong and Mao, 2018). In this study, the Cronbach’s *α* coefficient of the scale was 0.942.

#### Meaning in life

In this study, the Chinese version of the meaning in life scale compiled by Steger et al. (2006) and revised by Wang and Dai (2008) was used to test the subjects. A total of 10 topics, including a sense of meaning and seek a sense of meaning 2 dimensions, such as “I am looking for my life goal or mission,” “my life has no clear purpose” and so on. The scale uses Likert 7 points to score, from “completely inconsistent” to “completely consistent,” 1–7 points, respectively. The higher the score, the higher the meaning of life of the subjects. Studies have shown that the scale has good reliability and validity among Chinese college students (Zhu et al., 2022). In this study, the Cronbach *α* coefficient of the scale was 0.945.

#### Internet addiction

The Chinese version of Internet Addiction Scale revised by Young (2009) was adopted, there are 8 items in the scale, each item is answered with “yes” or “no.” The answer “no” is 1 point, and the answer “yes” is 2 points. The average score of the 8 items is calculated. The higher the score, the higher the tendency of internet addiction. The research has proved that the scale has good reliability and validity among Chinese college students (Cao et al., 2022). In this study, the Cronbach α coefficient of the scale was 0.907.

#### Suicidal ideation

The suicidal ideation scale was developed by You et al. (2014) was used to evaluate suicidal ideation. The scale consisted of two items (“Have you seriously considered ending your life before” and “Have you seriously considered ending your life in the past year”). Both items were scored by 3 points, 1 representing “never,” 2 representing “occasionally,” and 3 representing “often.” If and only if the subjects ‘scores on both items are 1, that is, the total score is 2, it means that they have no suicidal ideation, and the higher the score, the more serious the suicidal ideation. The scale showed high reliability and validity on Chinese college students (Wu et al., 2022). In this study, the Cronbach α coefficient of the scale was 0.874.

### Design and statistical analysis

Amos 26.0, SPSS26.0 and SPSS PROCESS plug-ins (Hayes, 2018) were used for data statistics and analysis. Firstly, Harman single factor test was used to test the common method deviation. Secondly, mean and standard deviation were used for descriptive statistics, and Pearson Correlation Coefficient was used to test the correlation between variables. Thirdly, We use Amos 26.0 to calculate fit indicators. Finally, taking exercise adherence as the independent variable, gender and grade as the control variable, suicide ideation as the dependent variable, meaning in life and internet addiction as the intermediary variable, the independent intermediary effect test was conducted using the Non-parametric Percentile Bootstrap Method (sample size 5,000, 95% confidence interval) and Model 4 in process, and the chain intermediary effect test was conducted with Model 6.

## Results

### Common method bias test

Harman single factor test was used to test the common method deviation. The results showed that there were 6 factors with characteristic roots greater than 1, and the cumulative variation of the first common factor explanation was 38.35%, which was less than the standard critical value of 40%, indicating that there was no serious common method bias in this study.

### Descriptive statistics and correlation analysis

As shown in Table 1, the correlation coefficients of exercise adherence, suicidal ideation, meaning in life and internet addiction were statistically significant. Correlation analysis showed that exercise adherence was negatively correlated with suicidal ideation and internet addiction, and positively correlated with meaning in life. Suicidal ideation was positively correlated with internet addiction and negatively correlated with meaning in life. Meaning in life was significantly negatively correlated with internet addiction. The relationship between variables supports the test of subsequent hypotheses.

**Table 1:** Means, standard deviations, and correlations among variables.

Variable	*M*	SD	Gender	1	2	3	4
Gender			1				
1. EA	21.71	5.664	0.008	1			
2. SI	2.37	0.952	0.041	−0.467^**^	1		
3. MIL	52.36	11.532	−0.101^**^	0.355^**^	−0.602^**^	1	
4. IA	1.094	0.223	0.02	−0.454^**^	0.772^**^	−0.622^**^	1

*N* = 1925. EA = exercise adherence, SI = suicidal ideation, MIL = meaning in life, IA = internet addiction. ^**^p < 0.01.

### Mediating effect test of meaning in life and internet addiction

SPSS Process model 6 combined with Bootstrap method was used to test the mediating effect of meaning in life and internet addiction between exercise adherence and suicidal ideation after 5,000 sampling and controlling for gender and age. The results of regression analysis showed (Table 2) that exercise adherence can significantly and positively predict suicide ideation (*β* = −0.467, *t* = −23.178, *p* < 0.01), and hypothesis 1 of this study was verified. Exercise adherence can significantly and positively predict the meaning in life (*β* = 0.355, *t* = 16.767, *p* < 0.01), meaning in life can significantly predict suicide ideation negatively (*β* = − 0.182, *t* = − 10.198, *p* < 0.01), exercise adherence can significantly negatively predict internet addiction (*β* = − 0.265, *t* = − 14.634, *p* < 0.01), internet addiction can significantly predict suicide ideation (*β* = 0.599, *t* = 32.112, *p* < 0.01), the meaning in life can significantly negatively predict internet addiction (*β* = − 0.531, *t* = − 29.181, *p* < 0.01).

**Table 2:** Analysis of regression relationship among variables.

Effect	Item	Effect	SE	*t*	*p*	LLCI	ULCI
Direct effect	EA ⇒ SI	−0.131	0.016	−8.361	< 0.01	−0.161	−0.100
Indirect Effect Process	EA ⇒ MLI	0.355	0.021	16.767	< 0.01	0.314	0.397
	EA ⇒ IA	−0.265	0.018	−14.634	< 0.01	−0.301	−0.230
	MIL⇒IA	−0.531	0.018	−29.181	< 0.01	−0.567	−0.496
	MIL⇒SI	−0.182	0.018	−10.198	< 0.01	−0.217	−0.147
	IA ⇒ SI	0.599	0.019	32.112	< 0.01	0.562	0.635
Total effect	EA ⇒ SI	−0.467	0.020	−23.178	< 0.01	−0.507	−0.428

EA = exercise adherence, SI = suicidal ideation, MIL = meaning in life, IA = internet addiction.

LLCI is the lower 95% limit for Bootstrap sampling and ULCI is the upper 95% limit for Bootstrap sampling.

It can be seen from the further Bootstrap test results (Table 3) that the total indirect effect value is − 0.336, accounting for 50.15% of the total effect value, and the confidence interval does not include 0, which indicates that the mediation effect between the meaning in life and internet addiction is significant between exercise adherence and suicide ideation. It consists of the following three paths:

**Table 3:** Mediation effect and effect size.

Path	Effect	Proportion of total (%)	95% confidence interval	
			Boot LLCI	Boot ULCI
EA → MIL→SI	−0.065	19.35	−0.086	−0.047
EA → IA → SI	−0.159	47.32	−0.190	−0.129
EA → MIL→IA → SI	−0.112	33.33	−0.141	−0.088
Total effect	−0.336		−0.393	−0.283

EA = exercise adherence, SI = suicidal ideation, MIL = meaning in life, IA = internet addiction.

Indirect effect 1, exercise adherence → meaning in life → suicide ideation, the effect value is − 0.065, accounting for 19.35% of the total effect value, the confidence interval does not include 0, and the indirect effect is significant. Hypothesis 2 is supported.

Indirect effect 2, exercise adherence → internet addiction → suicide ideation, the effect value is − 0.159, accounting for 47.32% of the total effect value, the confidence interval does not include 0, and the indirect effect is significant. Hypothesis 3 is verified.

**Figure 2:**
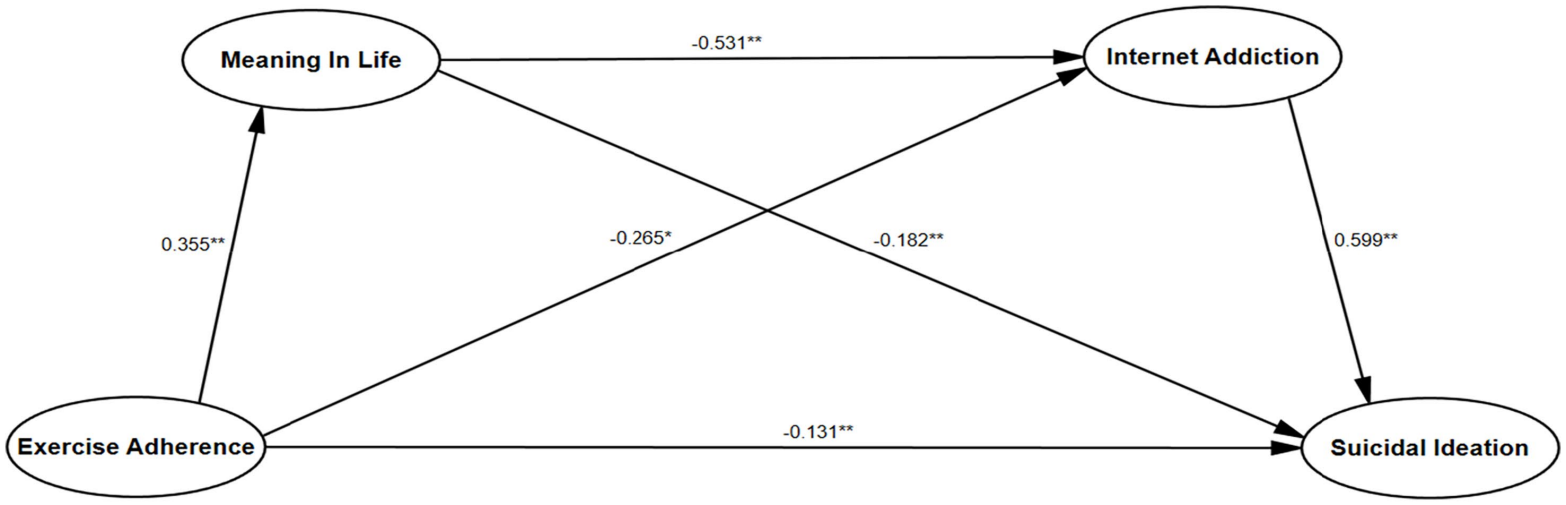
Chain mediating model of meaning in life and internet addiction between exercise adherence and suicidal ideation.

Indirect effect 3, exercise adherence → meaning in life → internet addiction → suicide ideation, the effect value is − 0.112, accounting for 33.33% of the total effect value, and the confidence interval does not include 0. The indirect effect of this path is significant. Hypothesis 4 of this study has also been verified. The mediating effect of meaning in life and internet addiction between exercise adherence and suicidal ideation is shown in Figure 2.

## Discussion

This study explored the relationship between exercise adherence and suicidal ideation of college students, as well as the mediating role of meaning in life and internet addiction. The results showed that exercise adherence could not only directly and negatively predict suicidal ideation, but also indirectly predict suicidal ideation through the separate mediating role of meaning in life and internet addiction, as well as the chain mediating role of the two. It further explains the reasons for the effect of exercise adherence on college students ‘suicidal ideation, which has certain enlightenment significance for inhibiting and preventing college students ‘suicidal ideation.

### Exercise adherence and suicidal ideation

This study confirmed that exercise adherence can significantly negatively predict suicidal ideation in college students. Hypothesis 1 is tested. The monoamine hypothesis holds that long-term physical exercise can not only promote brain microcirculation and improve the level of monoamine neurotransmitters in the brain, but also accelerate blood circulation and improve human metabolism, thus achieving the purpose of controlling individual adverse emotions (Wei and Yang, 2020). An important motivation for individuals to persist in physical exercise is to increase social opportunities, which can increase group identity and social reinforcement, and easily form social support networks with others, thus reducing the incidence of suicidal ideation. According to the Torsion Theory, psychological torsion does not necessarily lead to college students’ suicidal behavior, but the relationship between torsion and suicidal behavior may be regulated by social integration, social adjustment and psychological factors such as personality (Zhang et al., 2011). As an external protective factor, physical exercise can reduce the psychological torque of college students by playing the role of social integration or social regulation, thus affecting the generation of their suicidal ideation. According to the theory of sports psychology, physical exercise is conducive to generating positive emotions and alleviating negative emotions, which can promote the improvement of individual emotional regulation ability. Meanwhile, positive emotion management strategies help reduce the level of suicidal ideation of individuals (Lei et al., 2021). This suggests that, in the prevention and intervention of suicidal ideation in college students, exercise adherence can be regarded as a dominant external effective intervention factor into primary prevention measures, or it can be regarded as a relatively safe external protective factor to promote and pay attention to, so as to prevent the generation of suicidal ideation.

### Independent mediating effect of meaning in life

This study found that the meaning in life played a mediating role between exercise adherence and suicide ideation, which verified hypothesis 2. This is consistent with previous relevant research evidence, that is, exercise adherence significantly positively predicted meaning in life (Yu, 2018), and meaning in life negatively predicted suicide ideation (Zhang et al., 2022). This study takes three variables into consideration at the same time, revealing that exercise adherence is an important factor to promote the meaning in life, and also an important factor to prevent suicide ideation.

On the one hand, exercise adherence has a positive predictive effect on the meaning in life. The possible reasons are as follows: First, exercise adherence can not only improve the learning efficiency, social support and physical health of teenagers, but also affect the brain function through the brain nervous system, change depression, and effectively improve the meaning in life of individual life (Jiang et al., 2018). Second, physical exercise can promote the secretion of endorphins, make people feel happy and refreshed, and make individuals feel energetic, excited and full, so as to improve the meaning in life (Ding and Fan, 2002). Third, individuals with high exercise adherence can often achieve the established exercise goals, which is extremely beneficial to promote the individual’s sense of goal. In addition, good performance in competitive and cooperative exercise can also make individuals feel a sense of achievement and autonomy, which is conducive to improving the meaning in life.

On the other hand, meaning in life can negatively predicted suicidal ideation. Studies have shown that people with a high meaning in life have more expectations for life, and even when they encounter setbacks, they can also show strong psychological resilience in difficulties. They will not waver in their search for meaning of life due to the influence of external factors, and they will turn adversity, failure and temporary setbacks into action. This also indicates that the meaning in life has a positive effect on the prevention of suicidal ideation (Zhang et al., 2021). However, people with no meaning in life in their lives tend to suffer more psychological pain (Schulenberg, 2004). This may be because hope is an integral part of the meaning in life (Feldman and Snyder, 2005). When individuals lose their meaning in life, their goal-orientation will weaken and they will gradually lose hope for the future, low hope is associated with suicidal ideation to some extent (Snyder et al., 1991). Therefore, enhancing the meaning in life can reduce the suicidal ideation of college students.

### Independent mediating effect of internet addiction

This study also found that internet addiction plays a mediating role between exercise adherence and suicidal ideation, which verified hypothesis 3. This is consistent with previous relevant research evidence, that is, exercise adherence significantly negatively predicted internet addiction (Liu, 2018), and internet addiction significantly positively predicted suicide ideation (Wen et al., 2021).

This study confirmed that exercise adherence significantly negatively predicted internet addiction. The possible reasons are as follows: First of all, according to psychological theory, most college students with internet addiction have a certain degree of mental health disorders and cognitive disorders, and exercise adherence can improve individual psychological satisfaction (Teng, 2007), and promote the development of their socialization and cognitive ability (Yang and Xu, 2016), which is conducive to reducing internet addiction. Studies have shown that adolescent internet addiction is related to long-term depression, irritability, depression and other factors. Physical exercise can alleviate the confusion, anxiety, depression and other unhealthy mental states of internet addiction patients, so as to alleviate or even eliminate internet addiction (Liu, 2018). Secondly, according to the physiological theory, long-term physical exercise can make the exerciter’s pituitary gland secrete endorphins, which compete with addictive substances in the central nervous system for receptors, so that the individual produces a sense of happiness, and thus inhibits the attack of internet addiction (Jelena and Natasa, 2011).

In addition, this study found that internet addiction can predict suicidal ideation. The reasons are as follows: First, college students addicted to the internet are more likely to obtain information about suicide through relevant websites and forums. Secondly, according to the interpersonal theory of suicide, interpersonal psychology can predict and reveal suicidal ideation. Individuals with internet addiction spend too much time on the internet, take the network world as the real world, and are divorced from the times, have no common language with other people, and seriously lack social communication and interpersonal communication, resulting in loneliness and anxiety, depression, irritability and impulse and other adverse symptoms. Internet addicts not only damage their academic performance and interpersonal relationships, but also become restless when faced with more stressful life events, eventually leading to suicidal ideation and behavior (Pan et al., 2018).

### Chain mediating effect of meaning in life and internet addiction

This study confirmed the chain mediating effect of meaning in life and internet addiction, which is consistent with previous studies (Cao et al., 2022). Hypothesis 4 is verified. This study found that meaning in life can negatively predict internet addiction. The meaning in life is the perception of self-worth, individuals with low meaning in life are difficult to feel the value and meaning of their own existence in real life, and are prone to have a sense of boredom and confusion in life, as well as a great pressure and a psychology of escaping from reality. Online games and online social networking are the common ways for college students to reduce boredom and escape pressure today (Chen et al., 2019b). Therefore, college students with low meaning in life are more prone to internet addiction. Individuals with a high meaning in life are easy to find the meaning and goal of life, obtain the joy and spiritual enrichment of life, and experience positive emotions (Nie and Gan, 2017). Therefore, individuals with high meaning in life are not easy to cause internet addiction. In conclusion, the higher the exercise adherence of college students, the higher the meaning in life, the lower the internet addiction, and ultimately the lower the generation and development of suicidal ideation.

### Practical significance

This study examined the effect of exercise adherence on suicidal ideation, enriched the field of exercise adherence and suicidal ideation related research, to reduce college students suicidal ideation has a certain practical significance. First, exercise adherence is an important predictor of suicidal ideation. College students currently have a high suicide rate and should be given full attention. Suicide ideation is negatively correlated with individual sense of life meaning and positively correlated with internet addiction. Exercise adherence can not only positively predict sense of life meaning and negatively predict internet addiction, but also play an important role in predicting college students ‘suicidal ideation. Therefore, ensuring the adherence of college students ‘physical exercise should be an important part of college education. In improving the adherence of physical exercise, we should not only pay attention to the external environment factors, such as family sports environment, community sports environment and school sports environment, but also pay attention to the development of personality psychological characteristics, such as self-management, goal setting, self-monitoring. Teachers, especially physical education teachers, should create conditions for students to have good physical education and extracurricular physical exercise according to the characteristics of students’ physical and mental development, which can provide direct help for stimulating college students to adhere to physical exercise. Secondly, the sense of life meaning and internet addiction are important factors affecting college students ‘suicidal ideation. The mediating role of the sense of life meaning and internet addiction suggests that educators should pay attention to the influence of the sense of life meaning and internet addiction on college students ‘suicidal ideation. They should pay attention to improve the level of college students’ sense of life meaning and reduce internet addiction, improve the ability to deal with stressful events, treat problems with an objective and rational attitude, and actively respond to the pressure of life, study and work, so as to enhance the effect of exercise adherence on college students ‘mental health and reduce their suicidal ideation.

## Limitations and prospectives

Firstly, the conclusion of this study is based on the analysis of data. The collection of data comes from self-report. In the future, we can integrate various data collection methods to explore the impact of exercise adherence on suicidal ideation. Secondly, this study adopts a cross-sectional study design. In the future, the method of tracking research can be applied to deeply reveal the relationship between variables. Thirdly, this study found that meaning in life and internet addiction play a partial mediating role between exercise adherence and suicidal ideation. There may be other mediating variables in the relationship between the two. In the future, the comprehensive influence of multiple mediating variables can be considered. Fourthly, a consequence of the cross-sectional study design is the impossibility of determining the direction of effects between variables and confirming the presented conceptual model. Finally, according to the theory of meaning in life, the conceptual model can be: meaning in life → suicidal ideation. In this model, it may be meaningful to use exercise adherence and internet addiction as regulatory factors, as both factors are under the control of young people.

## Data availability statement

The original contributions presented in the study are included in the article/supplementary material, further inquiries can be directed to the corresponding authors.

## Ethics statement

This study was approved by the Research Ethics Committee of Zhaoqing University (No. 2022-1207-01). The collective test was adopted. Before the survey, it was explained to the students that the questionnaire was anonymously answered, emphasizing voluntary filling, and that the content is strictly confidential, and the results are only for scientific research. All questionnaires were collected on the spot. All subjects were informed of the purpose and characteristics of the study and signed informed consent.

## Author contributions

KG designed the study. ZX collected and analyzed the data, and wrote the manuscript. ZH and QM revised the manuscript. All authors contributed to the article and approved the submitted version.

## Funding

This research was funded by (1) 2022 Education Science Planning Project (Higher Education Special; grant number: 2022GXJK356); (2) 2021 Guangdong Undergraduate Teaching Quality and Teaching Reform Project—Teaching and Research Office of School Physical Education Curriculum Group, Serial number 96; (3) 2021 Zhaoqing University Quality Engineering and Teaching Reform project, grant number zlgc202111; and (4) Major Topics of Higher Education Scientific Research Plan in 2022, grant number 22JS0102.

## Conflict of interest

The authors declare that the research was conducted in the absence of any commercial or financial relationships that could be construed as a potential conflict of interest.

## Publisher’s note

All claims expressed in this article are solely those of the authors and do not necessarily represent those of their affiliated organizations, or those of the publisher, the editors and the reviewers. Any product that may be evaluated in this article, or claim that may be made by its manufacturer, is not guaranteed or endorsed by the publisher.
